# Vernacular Taxonomy, Cultural and Ethnopharmacological Applications of Avian and Mammalian Species in the Vicinity of Ayubia National Park, Himalayan Region

**DOI:** 10.3390/biology12040609

**Published:** 2023-04-17

**Authors:** Sayda Maria Bashir, Muhammad Altaf, Tanveer Hussain, Muhammad Umair, Muhammad Majeed, Wali Muhammad Mangrio, Arshad Mahmood Khan, Allah Bakhsh Gulshan, M. Haroon Hamed, Sana Ashraf, Muhammad Shoaib Amjad, Rainer W. Bussmann, Arshad Mehmood Abbasi, Ryan Casini, Abed Alataway, Ahmed Z. Dewidar, Mohamed Al-Yafrsi, Mahmed H. Amin, Hosam O. Elansary

**Affiliations:** 1 Department of Zoology, Women’s University of Azad Jammu and Kashmir, Bagh 12500, Pakistan; 2 Department of Forestry, Range and Wildlife Management, The Islamia University of Bahawalpur, Bahawalpur 63100, Pakistan; 3 College of Chemistry and Life Sciences, Zhejiang Normal University, Jinhua 321004, China; 4 Department of Botany, University of Gujrat, Hafiz Hayat Campus, Gujrat 50700, Punjab, Pakistan; 5 Department of Zoology, Faculty of Natural Sciences, Shah Abdul Latif University, Khairpur 66111, Pakistan; 6 Department of Botany, Government Hashmat Ali Islamia Associate College Rawalpindi, Rawalpindi 46300, Pakistan; 7 Department of Botany, Pir Mehr Ali Shah Arid Agriculture University Rawalpindi, Rawalpindi 46300, Pakistan; 8 Department of Botany, Ghazi University, Dera Ghazi Khan 32200, Punjab, Pakistan; 9 Department of Zoology, Wildlife and Fisheries, University of Agriculture, Faisalabad 38000, Pakistan; 10 Department of Zoology, University of Lahore, Sargodha 40100, Pakistan; 11 Department of Botany, Women’s University of Azad Jammu and Kashmir, Bagh 12500, Pakistan; 12 Department of Ethnobotany, Institute of Botany and Bakuriani Alpine Botanical Garden, Ilia State University, 0105 Tbilisi, Georgia; 13 Department of Botany, State Museum of Natural History, 76133 Karlsruhe, Germany; 14 Department of Environment Sciences, COMSATS University Islamabad, Abbottabad Campus, Abbottabad 22060, Pakistan; 15 School of Public Health, University of California, Berkeley, 2121 Berkeley Way, Berkeley, CA 94704, USA; 16 Prince Sultan Bin Abdulaziz International Prize for Water Chair, Prince Sultan Institute for Environmental, Water and Desert Research, King Saud University, Riyadh 11451, Saudi Arabia; 17 Department of Agricultural Engineering, College of Food and Agriculture Sciences, King Saud University, Riyadh 11451, Saudi Arabia; 18 Department of Plant Production, College of Food & Agriculture Sciences, King Saud University, Riyadh 11451, Saudi Arabia

**Keywords:** ethno-ornithological uses, ethno-mammalogical knowledge, biological diversity, Pakistan

## Abstract

**Simple Summary:**

Birds and mammals are strongly embedded in anthropological culture around the world. This study article discusses the cultural and therapeutic value of avian and mammalian species in the Ayubia National Park, KPK, Pakistan. To the best of our knowledge, this is the first quantitative study of the cultural utilization of avian and mammalian species in the studied region. This study’s ethno-biological findings indicate that the huge diversity of avian and mammalian species plays an essential role in the culture and health of native communities. These findings might aid in the long-term utilization of avian and mammalian species in the local healthcare system. For the sustainable utilization of avian and mammalian species, conservation efforts should be conducted with the involvement of conservation authorities.

**Abstract:**

Numerous investigations on plant ethnomedicinal applications have been conducted; however, knowledge about the medicinal use of wild animals is still limited. This present study is the second on the medicinal and cultural meaning of avian and mammalian species used by the population in the surrounding area of the Ayubia National Park, KPK, Pakistan. Interviews and meetings were compiled from the participants (*N* = 182) of the study area. The relative frequency of citation, fidelity level, relative popularity level, and rank order priority indices were applied to analyze the information. Overall, 137 species of wild avian and mammalian species were documented. Of these, 18 avian and 14 mammalian species were utilized to treat different diseases. The present research showed noteworthy ethno-ornithological and ethno-mammalogical knowledge of local people and their connection with fauna, which might be useful in the sustainable utilization of the biological diversity of the Ayubia National Park, Khyber Pakhtunkhwa. Furthermore, in vivo and/or in vitro examination of the pharmacological activities of species with the highest fidelity level (FL%) as well as frequency of mention (FM) might be important for investigations on faunal-based new drugs.

## 1. Introduction

Birds and mammals are important to human civilization. A variety of fauna is used in painting, medicine, music, food, literature, trade, export, hunting, poaching, magic, religion, and many other human expressions. Zootherapy is globally practiced and has deep historical origins [1]. Therapy contributes greatly to curing practices and magical healing rituals [2,3]. Ethnobiological studies serve to document this significant relationship [4], and relationships between local people and fauna must be taken into consideration [5], as we utilize fauna for ethnomedicine [2]. Animals and their derived products are not only utilized in ethnopharmacology, but they are also valuable as raw resources in the synthesis of allopathic medicines, with more than 8% of important chemicals gathered from fauna [6]. Despite their significance, studies on the uses of fauna have been rare when compared to ethnobotanical studies [2].

Nowadays, several studies involving indigenous people [7,8] have revealed that avian and mammalian species are the primary sources of protein throughout the world and across time, which is the main factor that motivates the trafficking, hunting, and massacre of these species. Hence, the ideal or optimal foraging theory, a model of evolutionary ecology that has been used in the research of human survival in numerous studies, were used in this study to investigate human populations’ preferences for medium- and large-sized avian and mammalian species. According to this notion, the fauna will attempt to consume as many resources as possible [9,10]. Studies have shown that when there is a dearth of the preferred fauna species, hunters must poach and hunt a greater number of less valuable fauna species, as well as dedicate more time to cover a larger area. Several cultural aspects must be considered in the selection, poaching, hunting, and utilization of fauna by local people since these have a significant influence on the populations of the fauna used [11,12]. In this context, knowledge of these matters is fundamental to supporting actions aimed at the conservation and management of the fauna utilized [8,13].

Records of folklore connected with medicinal and cultural uses of faunal species are necessary because many local societies are quickly losing their traditions and values [14]. In many traditional societies, fauna represent the main source of food and are used in medicine, entertainment, magic, research, culture, etc. [15,16,17,18]. A total of 195 mammalian species [19] and 688 avian species, for a total of 668, have been documented from Pakistan to date [20]. However, the cultural importance of fauna in Khyber Pakhtunkhwa has never been well documented, and the present study is expected to fill the gap regarding knowledge on the folklore value and therapeutic application of avian and mammalian species by the people of the Ayubia National Park, Khyber Pakhtunkhwa (KPK), Pakistan.

## 2. Materials and Methods

### 2.1. Study Area

**Figure 1:**
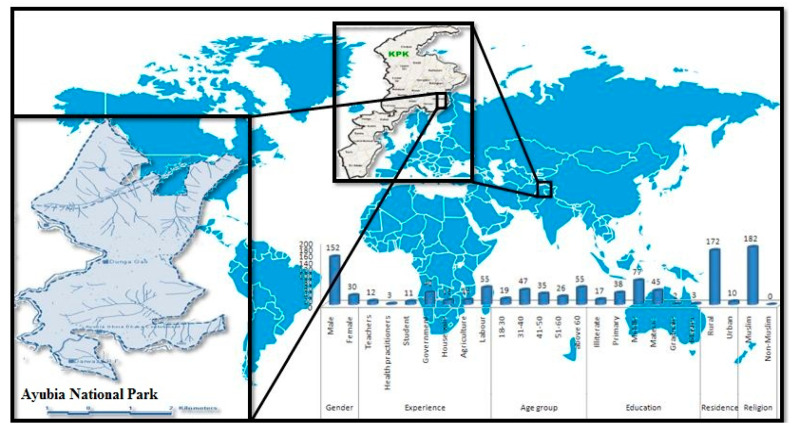
Map of the Ayubia National Park and ethnographic data of local informants.

**Figure 2:**
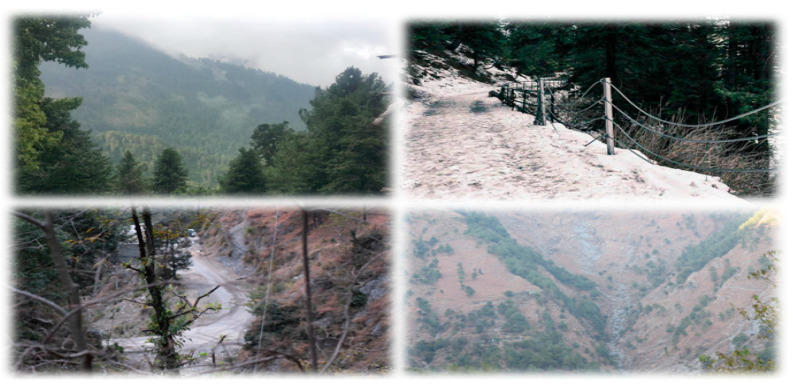
Landscapes of the study area.

The Ayubia National Park is located in the Reserved Forests of Galiat, North West Frontier Province (NWFP), Pakistan, between 73°22′54″ and 73°27′15″ E longitude and 34°00′48″ and 34°06′23″ N latitude (Figure 1). The total area of the national park is 33 km^2^, while the surrounding conserved woods are 150 km^2^. The park’s ecotypes include sub-alpine meadows, wet temperate woods, and sub-tropical pine forests (Figure 2). The park’s mission is to protect the rare plants and wildlife of the wet, temperate western Himalayan habitat.

### 2.2. Ethnography

Overall, 182 participants were selected through snowball sampling. Snowball sampling is useful for finding units to include when there is no clear list of the population you are interested in. Often, it is difficult to assess potential sampling error and make generalizations (i.e., statistical inferences) from the sample to the population [21]. We only included 30 women in our study due to cultural restrictions. The majority of the participants (*n* = 172) lived in rural areas, while 10 were settled in urban areas. Most of the informers belonged to the Mughal, Abbasi, Arain, Sheikh, Sayed, and Malik, which are the major ethnic groups of this study area. Most people spoke Hindko (90.6%), followed by Urdu (9%), Pahari (0.2%), and English (0.01%) (Figure 1).

### 2.3. Data Collection and Analysis

Surveys were carried out during 2017–2018, and data on folk medicine (traditional medicines) from avian species and mammalian species were gathered. Meetings and interviews were held with 182 informants (i.e., teachers, health practitioners, farmers, students, laborers, and housewives) after obtaining their verbal informed consent. Species were identified using the *Mammals of Ayubia National Park KPK* [22,23] and *Birds of Pakistan* [24,25].

### 2.4. Data Analysis

The data on ethnomedicinal applications and cultural values were examined using the following terms: relative frequency of citation, fidelity level, relative popularity level, and rank order priority.

### 2.5. Relative Frequency of Citation (RFC)

It was estimated using the equation described by J Tardío and M Pardo-de-Santayana [26], as follows:

RFC = FC/N (0 ≤ RFC ≥ 1).(1)

FC = Total number of informants for a folklore use of a specific species, and

N = Total number of informers.

### 2.6. Fidelity Level (FL)

The FL is the percentage of participants in the study area who claim to have used a specific type of species [27]. Its calculation was performed using the following formula [28]:

FL (%) = N_p_/FC × 100. (2)

where N_p_ is the number of major ailments of the informers for specific kinds of avian and mammalian species. FC = Frequency of citation for folklore use of a specific avian and mammalian species.

### 2.7. Relative Popularity Level (RPL)

The RPL is used to reflect the popularity of various species in the study area [29,30]. Avian and mammalian species were divided into two categories: unpopular and popular. The popular avian and mammalian species were cited for a higher proportion of the maximum FC. The remaining bird and mammalian species were considered unpopular. The FC mentioning avian and mammalian species for distinct folklore applications is shown on the *x*-axis, whereas the *y*-axis indicates the use of a number of diverse folklores for each bird and mammal species. A hypothetical horizontal line represents the average number of uses per avian and mammalian species independent of the FC. For popular avian and mammalian species, the relative popularity level was close to 1, whereas the relative popularity level was less than 1 for avian and mammalian species within the unpopular group.

### 2.8. Rank Order Priority (ROP)

The ROP index is used to appropriately arrange species, utilizing varying FL and RPL values as adjustment factors [31,32,33,34]. Rank order priority was used to rank the avian and mammalian taxa and was measured by the following equation [29,30]:

ROP = FL × RPL(3)

## 3. Results

### 3.1. Demography of Informants

A total of 182 participants, ranging in age from 18 to 60 years, were documented, as shown in Table 1. Maximum respondents (62%) were 41 to 60 years old. A total of 66% were literate with different levels of education, i.e., primary (38), middle (77), graduate (2), and master (3), as shown in Figure 1. One hundred seventy-two informers were from rural areas. The older participants held more traditional information compared to the younger participants.

### 3.2. Vernacular Taxonomy

Vernacular taxonomy refers to the native names of birds and mammals utilized for ethnomedicinal and folklore applications. Local names frequently include information related to the habitat, morphological differences, myths, and social associations. During the study, we noted that indigenous people identified 116 avian species.

**Figure 3:**
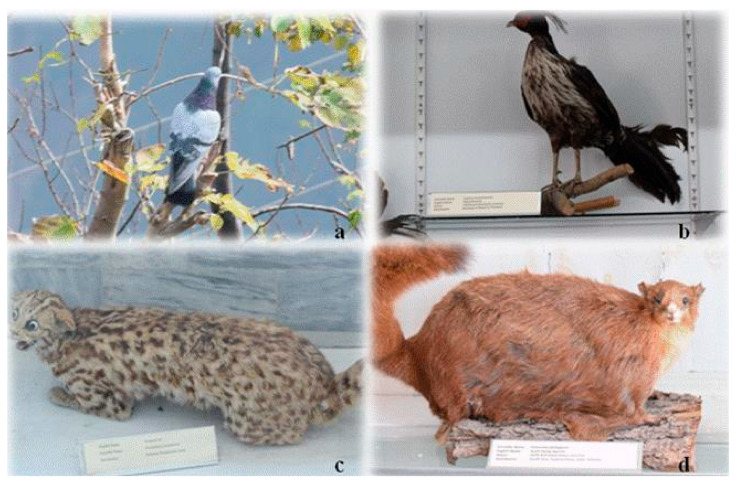
Significant species used in the study area: (**a**) Rock Pigeon, (**b**) Kalij Pheasant, (**c**) Leopard Cat, and (**d**) Giant Red Himalayan Flying Squirrel.

In the study area, the names of avian species were often associated with their voice, i.e., the brown-fronted woodpecker (tham thoka), oriental turtle dove (kogi), lesser cuckoo (koail, koel, and koal), common hoopoe (hud-hud), and spotted owlet (uloo). Vernacular bird names were also associated with a bird’s color, i.e., the small minivet (pelli chirri), long-tailed minivet (ratti chirri), and blue or Himalayan whistling thrush (nelli chiri). According to the informants, seven species of birds were locally known as baz, i.e., the northern goshawk (*Accipiter gentilis*), Eurasian sparrowhawk (*Accipiter nisus*), saker falcon (*Falco cherrug)*, peregrine falcon (*Falco peregrinus*), Hodgson or mountain hawk eagle (*Hieraaetus pennatus*), lesser spotted eagle (*Ictinaetus malayensis*), and common kestrel (*Nisaetus nipalensis*) (Figure 3).

Likewise, two birds (i.e., the white-cheeked tit, *Aegithalos leucogenys,* and the white-throated long-tailed tit, *Aegithalos niveogularis*) had the same local name, “pithpitha.” Similarly, four species, i.e., the short-eared owl (*Asio flanneus*), long-eared owl (*Asio otus*), mountain scops owl (*Otus spilocephalus*), and oriental scops owl (*Otus sunia*), had the same vernacular name, “ullo.” Similarly, ten species, such as the pink-browed rosefinch (*Carpodacus rodochroa*), brown dipper (*Cinclus pallasii*), blue robin (*Luseinia brunnea*), chestnut-bellied rock thrush (*Monticola rufiventris*), russet sparrow (*Passer rutilans*), coal tit (*Peraparus ater*), asian paradise flycatcher (*Terpsiphone paradise*), plain-backed thrush (*Zoothera mollissima*), oriental white-eye (*Zosterops palpebrosus*), and house sparrow (*Passer domesticus*) were all called “chirri.” Three species of crow, such as the carrion crow (*Corvus corone*), large-billed crow (*C. macrorhynchos*), and house crow (*C. splendens*), were called “kagh.” Five avian species, such as the brown-fronted woodpecker (*Dendrocopos auriceps*), rufous-bellied woodpecker (*D. hyperythrus*), fulvous-breasted woodpecker (*D. macei*), yellow-crowned woodpecker (*D. mahrattensis*), and grey-capped pygmy woodpecker (*D. nanus*), have the same vernacular name, “thum thoka.” Two species, i.e., *Pericrocotus ethologus* (long-tailed minivet) and *P. roseus* (rosy minivet), had the same vernacular name, “raja lal.” Finally, three avian species were known as “hirra” (the lesser sand plover, *charadrius mongolus*; large cuckooshrike, *Coracina macei*; and grey-winged blackbird, *Turdus boulboul*). English names were used for two species, i.e., the variable wheatear, *Oenanthe picata* (local name wheatear), and Asian koel, *Eudynamys scolopaceus* (local name koel).

It was documented that four species had more than one vernacular name, i.e., the common myna, *Acridotheres trusties* (gotari and myna), black drongo, *Dicrurus macrocercus* (kali chit and kalkalich), rosy minivet, *Pericrocotus roseus* (lambi dum and raja lal), and rufous-naped tit, *Periparus rufonuchalis* (pithpittha and pidda). Informants indicated that some species of birds received their name on the basis of color, e.g., the white-throated long-tailed tit, *Aegithalos niveogularis,* as “chitti pithpitha,” “chitti” meaning white; the brown dipper, *Cinclus pallasii,* as “bori chirri,” “bori” meaning brown. The Indian golden oriole, *Oriolus (oriouls) kundoo,* was known as “peeli chiri,” “peeli” meaning yellow; the grey-hooded warbler, *Phylloscopus xanthoschistos,* as “heri piddi,” “heri” meaning green; and the grey bush chat, *Saxicola ferreus,* as “salaiti chiri,” where “salaiti” means gray. The Asian paradise flycatcher, *Terpsiphone paradise,* as “bori-chiti chirri,” “brown-white.” The oriental white-eye, *Zosterops palpebrosus,* as “chiti akh chiri”, where “chiti” means white.

### 3.3. Cultural Uses and Folktales

The people in the study area had two narrative stories for avian and mammalian species, i.e., the house crow (*Corvus splendens*) was regarded as a wise animal, and the red fox (*Vulpes vulpes*) was regarded as a very clever animal. Four bird and mammal species were used for commercial purposes (sale), i.e., the peregrine falcon (*Falco peregrinus*), rose-ringed parakeet (*Psittacula krameri*), saker falcon (*Falco cherrug*), and Indian pangolin (*Manis crassicaudata*). Three species of birds were used as tools to capture small birds, i.e., the peregrine falcon (*Falco peregrinus*), the Hodgson or mountain hawk eagle (*Hieraaetus pennatus*), and the lesser spotted eagle (*Ictinaetus malayensis*). According to our informants, three avian and mammalian species were utilized for entertainment purposes, such as the rose-ringed parakeet (*Psittacula krameri*) because of its ability to speak and the mongoose because it can fight with snakes.

During the surveys, we noted that fourteen species were utilized as food, i.e., the jungle myna (*Acridotheres fuscus*), common myna (*Acridotheres tristis*), spotted dove (*Alectoris chukar*), common pigeon, (*Columba livia*), hill pigeon (*Columba rupestris*), kalij pheasant (*Lophura leucomelanos*), house sparrow (*Passer domesticus*), russet sparrow (*Passer rutilans*), koklass pheasant (*Pucrasia macrolopha*), oriental turtle dove, (*Streptopelia oriental*), drongo cuckoo (*Surniculus lugubris*), jungle babbler (*Turdoides striata*), chukar partridge, (*Atectoris chukar*), and cheer pheasant (*Catreus wallichii*). In the vicinity of the National Park, people only ate specific birds, obeying Islamic rules. Certain birds and mammals were used as food (Table 1), although Islam forbids consuming “insectivores,” “scavengers,” “carnivores,” and “piscivores.”

During research, we noted that four avian species were regarded as harmful for chickens and pet animals, such as the black eagle (*Buteo rufinus*), long-legged buzzard (*Buteo teesa*), Hodgson or mountain hawk eagle (*Hieraaetus pennatus*), and saker falcon (*Falco cherrug*). Moreover, fourteen species of mammals were regarded as harmful, including the common leopard, red fox, Asiatic jackal, and leopard cat, which are harmful to livestock; the house shrew and house mouse damage clothes and household items. The giant red Himalayan flying squirrel, Royle’s pika, small Kashmir flying squirrel, northern palm squirrel, Indian mole rat, Indian crested porcupine, rhesus monkey, Indian wild boar, Asiatic jackal, and red fox because they were suspected of damaging crops.

According to respondents, four species of birds were used in magic i.e., *Asio flammeus, Asio otus, Otus spilocephalus and Otus sunia*. They were used in black magic for evil purposes. Local people also used two mammalian species for magic: the presence of *quills* (Indian crested porcupine) created disgust among people, and the scales of the Indian pangolin were known as a symbol of health.

In the study area, six birds and one mammal were exported illegally: chukar partridge (*Atectoris chukar*), common kestrel (*Falco tinnunculus*), Hodgson or mountain hawk eagle (*Hieraaetus pennatus*), lesser spotted eagle (*Ictinaetus malayensis*), saker falcon (*Falco cherrug*), peregrine falcon (*Falco peregrinus*), and Indian pangolin (*Manis crassicaudata*). During the surveys, we noted that all avian species and two mammalian species, i.e., the common leopard (*Panthera pardus*) and leopard cat (*Prionailurus bengalensis*), were used as decoration for ornamental purposes.

### 3.4. Body Parts Used

**Figure 4:**
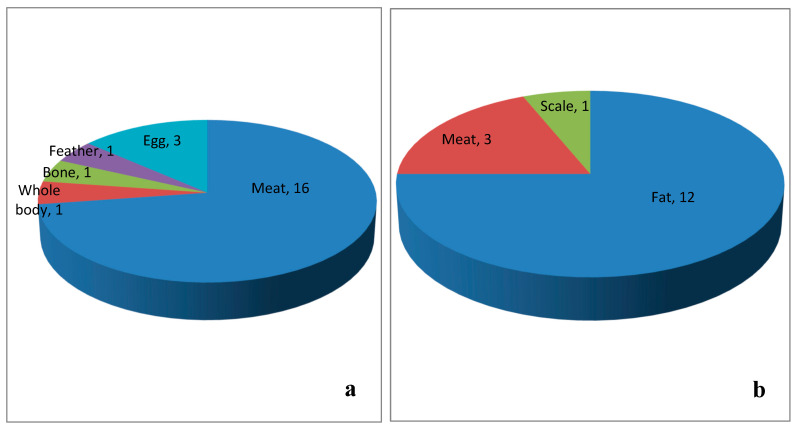
(**a**) Birds (**b**) and mammal parts used against different diseases in the study area.

Meat of the avian taxa was the most highly consumed body part (16 medications), followed by eggs (3), the whole body (1), feathers (1), and bones (1), as shown in Figure 4a.

Local people utilized the meat of common hoopoe, common myna, common pigeon, green shrike-babbler, greenish warbler, grey-hooded warbler, hill pigeon, house sparrow, jungle babbler, jungle myna, kalij pheasant, koklass pheasant, mistle thrush, oriental turtle dove, russet sparrow, and spotted dove to treat anemia, bronchitis, epilepsy, fever, infertility, kidney problems, low blood pressure, maturity in girls, puberty in young girls, menorrhagia, paralysis, skin diseases, weakness, and whooping cough. Similarly, the eggs of the hill pigeon, common pigeon, and house sparrow were used to treat anemia, bronchitis, epilepsy, fever, infertility, low blood pressure, menorrhagia, paralysis, puberty in young girls, and weakness. Likewise, the feathers of the mistle thrush were used to treat skin diseases, and the bones of the house crow were used to treat ear infections (Table 2).

**Figure 5:**
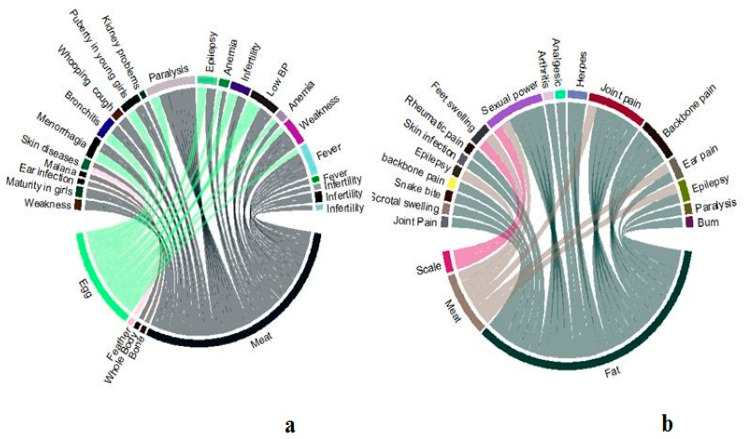
Chords for bird (**a**) and mammalian (**b**) species.

In mammalian species, fats were the most consumed part of the body (12 recipes), followed by meat (3) and scale (1) (Figure 5). People in the study area used the fat of the house shrew, house mouse, Indian crested porcupine, leopard cat, small Indian mongoose, Indian wild boar, small Kashmir flying squirrel, giant red Himalayan flying squirrel, Indian grey mongoose, Himalayan palm civet, and common leopard as an analgesic and for arthritis, backbone pain, burns, herpes, joint pain, paralysis, rheumatic pain, scrotal swelling, sexual power, skin infection, and snake bites. Local people applied the meat of the Northern palm squirrel, Indian pangolin, and red fox to treat epilepsy, feet swelling, sexual power, ear pain, and joint pain. Inhabitants used the scales of Indian pangolins to treat foot swelling and enhance sexual power (Table 2).

### 3.5. Quantitative Analysis

#### 3.5.1. Frequency of Citation (FC)

The highest FC was recorded for the common myna (81), followed by the house sparrow (FC = 62), the kalij pheasant, and the koklass pheasant, while the lowest citation (FC = 1) was for the greenish warbler.

#### 3.5.2. Fidelity Level (FL)

**Figure 6:**
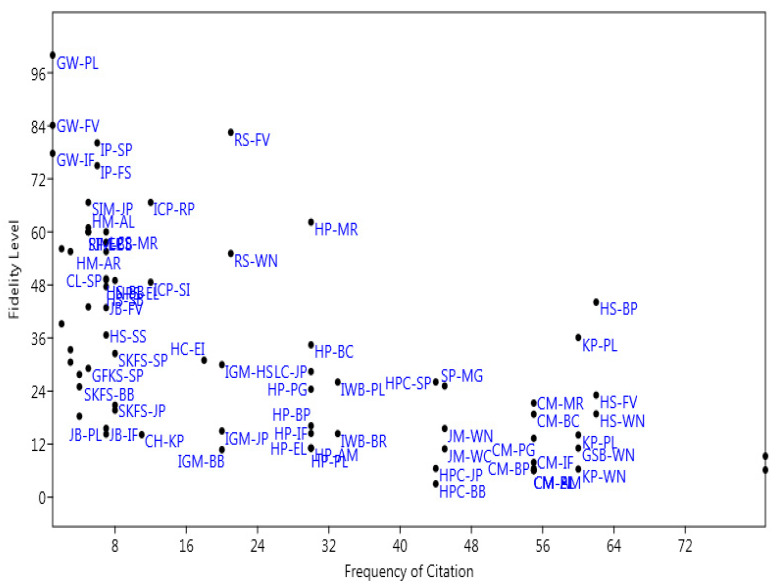
Scatter plot showing the fidelity level (FL%) of species with frequency of citation (FC); circled code shows the mammal and bird names as given in Table 2.

The FL is utilized to recognize diseases (treated with avian and mammalian parts) that are most liked by the people for the healing and curing of sicknesses. Avian and mammalian species with the most therapeutic uses in the study area have the greatest FL. In our research, the FL of diseases cured by avian and mammalian species varied from 2.27 percent to 100 percent (Table 2 and Figure 6). GW-FV (fever cured with greenish warbler), GW-PL (paralysis cured with greenish warbler), GW-IF (infertility cured with greenish warbler), and KP-MG (maturity in girls cured with greenish warbler) reached 100% FL, while HPC-JP (joint pain cured with Himalayan palm civet) and HPC-BB (backbone pain cured with Himalayan palm civet) had the lowest (27%) (Table 2).

#### 3.5.3. Relative Popularity Level (RPL)

The RPL of bird and mammalian species (Table 2) was analyzed, and seven taxa with the highest significance were added for additional debate. The CM-MR, CM-BC, CM-PG, CM-PL, CM-EL, CM-AM, CM-IF, CM-BP, KP-WN, KP-PL, HS-WN, LC-JP, GSB-WN, KP-PL, and HS-FV have an RPL value of 1.0. The lowest RPL values were recorded for GW-FV, GW-PL, and GW-IF (RPL = 0.02).

#### 3.5.4. Rank Order Priority (ROP)

ROP is used to number the species according to their FL values. The measured level of ROP for each avian and mammalian species is documented in Table 2. The ROP of three avian and mammalian species out of 32 was above 28. The HS-WN, HP-BC, and CM-BC were highly utilized, with ROP values of 32.26, 27.78, and 25.45, respectively.

#### 3.5.5. Principal Component Analysis (PCA)

**Figure 7:**
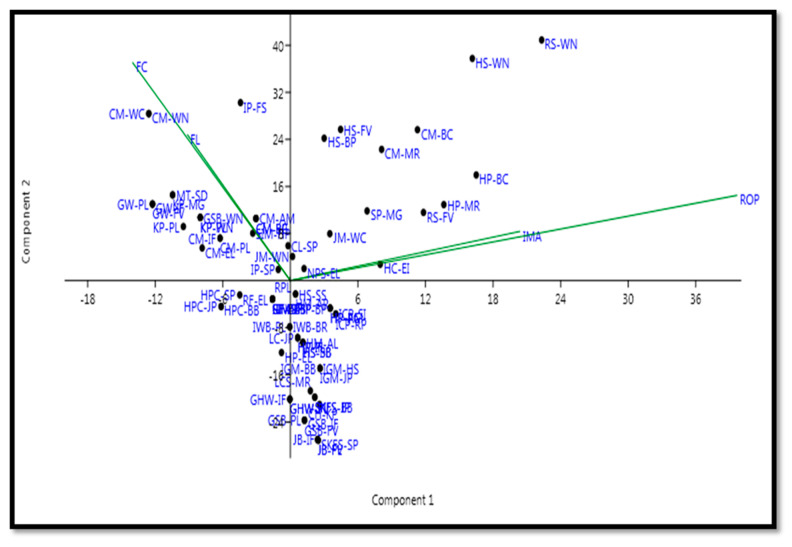
Principal component analysis is used to analyze ethnomedicinal data from mammalian and avian species. The locations of the arrows in relation to components 1 and 2 demonstrate the degree of correlation between the independent variables (FC, IMA, FL, RPL, and ROP).

**Figure 8:**
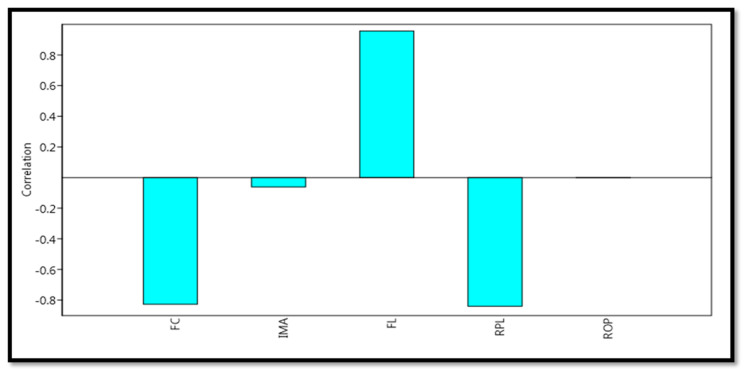
Correlation among ethno-variables, i.e., rank order priority (ROP), frequency of citation (FC), informant of major ailment (IMA), fidelity level (FL), and relative popularity level (RPL).

PCA is used to analyze ethnomedicinal data from mammalian and avian species, allowing for plot ordination in terms of three variables. The following variables were included: frequency of citation (FL), informant of major ailment (IMA), fidelity level (FL), relative popularity level (RPL), and rank order priority (ROP). The PCA result gave a sum of all eigenvalues with a total inertia of 1456.85. The first eigenvalue was high (1148.31), indicating a strong gradient in the distribution of indigenous knowledge along the first axis (PC1). Figure 7 displays that the first two axes of the principal component analysis reveal 96.9% variance in the data (component 1: 78.821%; component 2: 18.074%). The variables, i.e., FC (*r* = −0.55473), informant of major ailment (*r* = −0.0074), FL (*r* = 0.83195), RPL (*r* = −0.00935), and ROP (*r* = −0.00044) were correlated with the first axis (component 1), while FC (*r* = 0.77802), informant of major ailment (*r* = 0.17562), FL (*r* = 0.52063), RPL (*r* = 0.011839), and ROP (*r* = 0.30438) were positively correlated with component 2 as shown in Figure 8.

## 4. Discussion

Meat contains nitrogenous and non-nitrogenous substances and other components [35,36]. Poultry, cattle, sheep, goats, pork, and fish are the most common meat sources globally. However, in a few nations, particularly in semiarid and arid areas, the meat of the camel is renowned as the primary supply of protein, equaling and, in some cases, exceeding the commercial importance of other meats [37,38,39,40]. Meats of different species, i.e., *Acridotheres ginginianus*, *Acridotheres tristis, Ammoperdix heyi*, *Anas platyrhynchos*, *Aratinga cactorum*, *Bagarius bagarius*, *Bos taurus*, *Bubalus bubalis*, *Calotes versicolor*, *Camelus dromedaries*, *Capra aegagrus hircus*, *Channa marulius*, *Cirrhinus mrigala*, *Columba livia*, *Columba rupestris*, *Coturnix coturnix*, *Ctenopharyngodon idella*, *Cyprinus carpio*, *Dicrurus macrocercus*, *Egretta garzetta*, *Equus caballus*, *Eudynamys scolopaceus, Francolinus francolinus*, *Funnambulus pennant*, *Gallus gallus domesticus*, *Hystrix indica*, *Labeo rohita*, *Lepidocephalichthys thermalis*, *Lepus nigricollis*, *Macaca mulatta*, *Mimus saturninus*, *Nothura boraquira*, *Oreochromis niloticus*, *Ovis aries*, *Passer cinnamomeus, Passer domesticus, Pterocarpus giganteus*, *Rana clamitans, Rita rita*, *Serpentes* spp., *Spilopelia chinensis*, *Streptopelia orientalis*, *Suncus murinus*, *Trochalopteron lineatum*, *Upupa epops*, *Vulpes vulpes*, and *Wallago attu* are utilized in different folk therapies to treat the following: allergies, anemia, asthma, diabetes, diarrhea, dysentery, epilepsy, eye problems, fever, flu, hemoglobin, hepatitis, jaundice, kidney problems, muscular pain, paralysis, paralysis, scorpion bite, sore throats, tuberculosis, and whooping cough [41,42,43,44,45,46,47,48,49,50,51,52,53,54,55,56,57,58,59,60,61,62,63,64].

Ethnozoologists discovered that various species of animals, including the Indian gagata, horse, goat, fruit bat, crab-eating macaque, common carp, deer, crow, cinereous vulture, and alpine musk deer, were utilized to cure a variety of ailments, including wound healing, urine problems, heart strength, ear aches, chest pain, lumbago, and skin issues [41,47,61,62,65,66,67,68,69,70].

Ethnobiologists discovered that fats are consumed to restore health and treat nerve problems and aging issues [71,72,73,74,75,76,77,78]. The previous published data showed that the fats of various animal species, i.e., the wild boar, turtle, sheep, mongoose, bat, lizard, dolphin, Indus Valley spiny-tailed ground lizard, Indian rock python, Indian flap-shelled turtle, Indian bullfrog, horse, Himalayan serow, jackal, hare, green pond frog, goat, deer, cow, common leopard gecko, and Asiatic black bear, are used to cure different ailments, such as wounds [79,80], back pain [80,81], sexual problems [47,62,66,80,82,83,84], impotency [62,66], muscle pain, ear disease [65,82], cancer [62], arthritis [80], paralysis, [41,80], allergies [41,67,80], typhoid [47,59], and joint pain [83]. Eggs are a good source of nutrients. Breast cancer, bronchitis, asthma, high blood pressure, burns, diabetes, eye pain, CNS, cold, fever, jaundice, nourishment, night blindness, sprains, weak weight loss, and weakness are all cured by eggs [3,17,49,50,57,66,79,80,85,86,87,88,89,90,91,92,93,94,95,96,97]. Eggs are an incredibly tasty and healthful item that can be utilized in a variety of ways [97]. Eggs are composed of components that provide the best environment for an embryo’s development and growth. With the exception of vitamin C, eggs are a major source of important nutrients.

Avian feathers are applied in folk therapy to heal asthma, cough, flu, typhoid, and headaches [47,50,52,79,88,92,96,98,99,100,101,102]. Bone is composed of collagen [103], water, lipids [104,105], noncollagenous proteins [106,107], and minerals [108,109]. Lipids make up less than 2% of bone mass [104,105]. Different ethnobiologists noted that faunal species, i.e., alpine musk deer (*Moschus chrysogaster*), cinereous vulture (*Aegypius monachus*), common carp (*Cyprinus carpio*), crab-eating macaque (*Macaca fascicularis*), crow (*Corvus* spp.), deer (*Cervus* spp.), fruit bat (*Pteropus* spp.), goat (*Capra aegagrus*), horse (*Equus ferus caballus*), Indian gagata (*Gagata cenia*), and pigs (*Sus* spp.) are used for different ailments, such as improving chest pain [68], cough [47], digestion [65], ear aches [47,80], eyesight [68], heart strength [65], lumbago [110], neuralgia [41], skin [67], urine problems [62], and wounds [66].

## 5. Conclusions

The residents of our study region have deep ties to local wildlife and have vital traditional knowledge about bird and mammalian species. Folklore of different avian and mammalian taxa was documented, mainly to conserve their traditional knowledge and cultural usage among the indigenous peoples in the vicinity of the Ayubia National Park, KPK, Pakistan. In this study, the ethnopharmacological and folklore applications of 18 bird species as well as 14 mammalian taxa were documented for the first time. Feathers are utilized as biomaterials since they are inexpensive and environmentally beneficial, and they are applied in an ornamental way as well as in playthings. Bones contain up to 95% protein, fibers, and minerals, such as phosphate and calcium, which help prevent bone fracture. This knowledge is useful for contemporary pharmaceutical research since it may open up opportunities for the identification of new compounds with significant therapeutic potential in the future. Important toxicological studies would be necessary to guarantee the continuous and secure use of the presented practices. Local communities and responsible bodies must conserve medicinal plants to avoid further losses.

## Figures and Tables

**Table 1:** Cultural uses of birds and mammals in the study area.

Sr. No.	Scientific Name	Species Authority	Common Name	Vernacular Name	Status	MD	NR	CC	TL	ET	FD	HF	MG	EX	OR	FC	RFC	CU
Birds																		
1	*Accipiter gentilis*	Linnaeus, 1758	Northern Goshawk	Baz	LC	×	×	×	×	×	×	×	×	×	҂	3	0.002	1
2	*Accipiter nisus*	Linnaeus, 1758	Eurasian Sparrow Hawk	Chirimar Baz	LC	×	×	×	×	×	×	×	×	×	҂	1	0.001	2
3	*Acridotheres fuscus*	Wagler, 1827	Jungle Myna	Gotari	LC	҂	×	×	×	×	҂	×	×	×	҂	45	0.030	8
4	*Acridotheres tristis*	Linnaeus, 1766	Common Myna	Gotari, Myna	LC	҂	×	×	×	×	҂	×	×	×	҂	81	0.068	10
5	*Aegithalos leucogenys*	Gould, 1855	White-cheeked Tit	Chitti Pithpitha	LC	×	×	×	×	×	×	×	×	×	҂	3	0.002	2
6	*Aegithalos niveogularis*	Gould, 1855	White-throated Long-tailed Tit	Pithpitha	LC	×	×	×	×	×	×	×	×	×	҂	1	0.001	1
7	*Alectoris chukar*	Linnaeus, 1758	Chukar Partridge	Chakore	LC	×	×	×	×	×	҂	×	×	҂	҂	2	0.001	1
8	*Asio flammeus*	Pontoppidan, 1763	Short-eared Owl	Uloo	LC	×	×	×	×	×	×	×	҂	×	҂	2	0.001	2
9	*Asio otus*	Linnaeus, 1758	Long-eared Owl	Uloo	LC	×	×	×	×	×	×	×	҂	×	҂	2	0.001	2
10	*Buteo rufinus*	Garnot, 1828	Black Eagle	Basha, Baz	LC	×	×	×	×	×	×	҂	×	×	҂	1	0.001	1
11	*Buteo teesa*	Franklin, 1831	Long-legged Buzzard	Chitti aankh wala Baz	LC	×	×	×	×	×	×	҂	×	×	҂	3	0.002	1
12	*Carpodacus rodochroa*	Vigors, 1831	Pink-browed Rosefinch	Gulabi Chirri	LC	×	×	×	×	×	×	×	×	×	҂	10	0.007	1
13	*Catreus wallichii*	Hardwicke, 1827	Cheer Pheasant	Jungli kukar	LC	×	×	×	×	×	҂	×	×	×	҂	45	0.030	1
14	*Certhia himalayana*	Vigors, 1832	Bar-tailed Treecreeper	Chirii	LC	×	×	×	×	×	×	×	×	×	҂	2	0.001	1
15	*Chaimarrornis leucocephalus*	Vigors, 1831	White-capped Red Start	Thirkara	LC	×	×	×	×	×	×	×	×	×	҂	2	0.001	1
16	*Charadrius mongolus*	Pallas, 1776	Lesser Sand Plover	Chirra	LC	×	×	×	×	×	×	×	×	×	҂	1	0.001	1
17	*Cinclus pallasii*	Temminck, 1820	Brown Dipper	bori chirri	LC	×	×	×	×	×	×	×	×	×	҂	6	0.004	2
18	*Columba livia*	Gmelin, 1789	Common Pigeon	Jungli kubutar	LC	҂	×	×	×	×	҂	×	×	×	҂	55	0.037	24
19	*Columba rupestris*	Pallas, 1811	Hill Pigeon	Kbuter	LC	҂	×	×	×	×	҂	×	×	×	҂	30	0.020	24
20	*Coracina macei*	Lesson, 1831	Large Cuckooshrike	Nella Chirra	LC	҂	×	×	×	×	×	×	×	×	҂	7	0.005	2
21	*Corvus corone*	Linnaeus, 1758	Carrion Crow	Kagh	LC	×	×	×	×	×	×	×	×	×	҂	100	0.068	2
22	*Corvus macrorhynchos*	Wagler, 1827	Large-billed Crow	Jungli kagh	LC	×	×	×	×	×	×	×	×	×	҂	90	0.061	1
23	*Corvus splendens*	Vieillot, 1817	House Crow	Kagh	LC	҂	҂	×	×	×	×	×	×	×	҂	7	0.123	4
24	*Cuculus canorus*	Linnaeus, 1758	Common Cuckoo	Koail	LC	×	×	×	×	×	×	×	×	×	҂	3	0.002	1
25	*Cuculus poliocephalus*	Latham, 1790	Lesser Cuckoo	Coail	LC	×	×	×	×	×	×	×	×	×	҂	2	0.001	1
26	*Dendrocitta vagabunda*	Latham, 1790	Rafous Treepie	Bara lam dumbara Chinjar	LC	×	×	×	×	×	×	×	×	×	҂	55	0.037	1
27	*Dendrocopos auriceps*	Vigors, 1831	Brown-fronted Woodpecker	Thum thoka	LC	×	×	×	×	×	×	×	×	×	҂	26	0.018	2
28	*Dendrocopos hyperythrus*	Vigors, 1831	Rufous-bellied Woodpecker	Thum thoka	LC	×	×	×	×	×	×	×	×	×	҂	30	0.020	2
29	*Dendrocopos macei*	Linnaeus, 1758	Fulvous-breasted Woodpecker	Thum thoka	LC	×	×	×	×	×	×	×	×	×	҂	2	0.001	2
30	*Dendrocopos mahrattensis*	Latham, 1801	Yellow-crowned Woodpecker	Thum thoka	LC	×	×	×	×	×	×	×	×	×	҂	3	0.002	1
31	*Dendrocopos nanus*	Blyth, 1845	Grey-capped Pygmy Woodpecker	Thum thoka	LC	×	×	×	×	×	×	×	×	×	҂	2	0.001	3
32	*Dicrurus leucophaeus*	Vieillot, 1817	Ashy Drongo	Kalchit	LC	×	×	×	×	×	×	×	×	×	҂	77	0.052	2
33	*Dicrurus macrocercus*	Vieillot, 1817	Black Drongo	Kali chit, Kalkalich	LC	×	×	×	×	×	×	×	×	×	҂	30	0.020	1
34	*Emberiza melanocephala*	Scopoli, 1769	Black-headed Bunting	Boli	LC	×	×	×	×	×	×	×	×	×	҂	2	0.001	2
35	*Eudynamys scolopaceus*	Linnaeus, 1758	Asian Koel	Koel	LC	×	×	×	×	×	×	×	×	×	҂	10	0.007	2
36	*Eumyias thalassinus*	Swainson, 1838	Verditer Flycatcher	Tik-tiki	LC	×	×	×	×	×	×	×	×	×	҂	21	0.014	1
37	*Euodice malabarica*	Linnaeus, 1758	Indian Silverbill	Slaiti chny chiri	LC	×	×	×	×	×	×	×	×	×	҂	3	0.002	1
38	*Falco cherrug*	Gray, 1834	Saker Falcon	Baz	LC	×	×	҂	×	×	×	҂	×	҂	҂	1	0.001	2
39	*Falco peregrinus*	Tunstall, 1771	Peregrine Falcon	Baz	LC	×	×	҂	҂	×	×	×	×	҂	҂	2	0.001	4
40	*Falco tinnunculus*	Linnaeus, 1758	Common kestrel	Baz	LC	×	×	×	×	×	×	×	×	×	҂	1	0.001	1
41	*Ficedula superciliaris*	Jerdon, 1840	Ultramarine Flycatcher	Tik-tikii	LC	×	×	×	×	×	×	×	×	×	҂	2	0.001	1
42	*Garrulax erythrocephalus*	Vigors, 1832	Himalayan Laughingthrush	Sorh	LC	×	×	×	×	×	×	×	×	×	҂	4	0.003	1
43	*Garrulax rufogularis*	Lesson, 1831	Rufous-chinned Laughingthrush	Chinny wali chirii	CR	×	×	×	×	×	×	×	×	×	҂	3	0.002	1
44	*Garrulax variegatus*	Vigors, 1831	Variegated Laughingthrush	Chirii	LC	×	×	×	×	×	×	×	×	×	҂	2	0.001	1
45	*Garrulus glandarius*	Linnaeus, 1758	Eurasian Jay	Rollar	LC	×	×	×	×	×	×	×	×	×	҂	5	0.003	1
46	*Gyps fulvus*	Hablizl, 1783	Eurasian Griffon Vulture	Gid	LC	×	×	×	×	×	×	×	×	×	҂	2	0.001	2
47	*Gyps himalayensis*	Hume, 1869	Himalayan Vulture	Gadh	NT	×	×	×	×	×	×	×	×	×	҂	1	0.001	1
48	*Hieraaetus pennatus*	Gmelin, 1788	Hodgson or Mountain Hawk Eagle	Baz	LC	×	×	×	҂	×	×	҂	×	×	҂	2	0.001	4
49	*Hirundapus caudacutus*	Latham, 1801	White-throated Needletail	Lambi dum chiri	LC	×	×	×	×	×	×	×	×	×	҂	2	0.001	1
50	*Hirundo smithii*	Leach, 1818	Wire-tailed Swallow	Ababeel	LC	×	×	×	×	×	×	×	×	×	҂	2	0.001	1
51	*Hypsipetes Leucocephalus*	Gmelin, 1789	Black Bulbul	Bulbull	LC	×	×	×	×	×	×	×	×	×	҂	5	0.003	1
52	*Ictinaetus malayensis*	Temminck, 1822	Lesser spotted Eagle	Baz	LC	×	×	×	҂	×	×	҂	×	҂	҂	1	0.001	5
53	*Lanius Isabellinus*	Ehrenberg, 1833	Isabelline Shrike	Latora	LC	×	×	×	×	×	×	×	×	×	҂	2	0.001	1
54	*Lanius phoenicuroider*	Schalow, 1875	Red-tailed Shrike	Latore	LC	×	×	×	×	×	×	×	×	×	҂	2	0.001	1
55	*Lophophorus impejanus*	Latham, 1790	Himalayan Monal	Monul	LC	×	×	×	×	×	×	×	×	×	҂	2	0.001	1
56	*Lophura leucomelanos*	Latham, 1790	Kalij pheasant	Bun kukar	LC	҂	×	×	×	×	҂	×	×	×	҂	60	0.041	3
57	*Lucinia brunnea*	Hodgson, 1837	Blue robin	Nelli chirri	LC	×	×	×	×	×	×	×	×	×	҂	2	0.001	2
58	*Milvus migrans*	Boddaert, 1783	Black kite	Cheel	LC	×	×	×	×	×	×	×	×	×	҂	1	0.001	1
59	*Monticola rufiventris*	Jardine and Selby, 1833	Chestnut-bellied Rock Thrush	Niki chirri	LC	×	×	×	×	×	×	×	×	×	҂	2	0.001	2
60	*Mycerobas melanozanthos*	Hodgson, 1836	Spot-winged Grosbeak	Chnar	LC	×	×	×	×	×	×	×	×	×	҂	1	0.001	1
61	*Myophonus caeruleus*	Scopoli, 1786	Blue or Himalayan Whistling Thrush	Pahari, Kholchora	LC	×	×	×	×	×	×	×	×	×	҂	5	0.003	2
62	*Neophron percnopterus*	Linnaeus, 1758	Egyptian vulture	Gid	EN	×	×	×	×	×	×	×	×	×	҂	1	0.001	1
63	*Nisaetus nipalensis*	Hodgson, 1836	Mountain Hawk Eagle	Baz	LC	×	×	×	×	×	×	҂	×	҂	҂	2	0.001	1
64	*Oenanthe picata*	Blyth, 1847	Variable Wheatear	Wheater	LC	×	×	×	×	×	×	×	×	×	҂	4	0.003	1
65	*Oriolus (oriouls) kundoo*	Sykes, 1832	Indian Golden Oriole	Peeli chiri	LC	×	×	×	×	×	×	×	×	×	҂	1	0.001	1
66	*Otus spilocephalus*	Blyth, 1846	Mountain Scops Owl	Uloo	LC	×	×	×	×	×	×	×	҂	×	҂	4	0.003	2
67	*Otus sunia*	Hodgson, 1836	Oriental Scops Owl	Uloo	LC	×	×	×	×	×	×	×	҂	×	҂	1	0.001	2
68	*Parus magor*	Linnaeus, 1758	Great Tit	Pithpitta	LC	×	×	×	×	×	×	×	×	×	҂	3	0.002	2
69	*Parus monticolus*	Vigors, 1831	Green-backed Tit	Pithpittha	LC	×	×	×	×	×	×	×	×	×	҂	1	0.001	1
70	*Parus xanthogenys*	Vigors, 1831	Black-lored Tit	Pithpittha	LC	×	×	×	×	×	×	×	×	×	҂	2	0.001	1
71	*Passer domesticus*	Blyth, 1849	House Sparrow	Chirri	LC	҂	×	×	×	×	҂	×	×	×	҂	62	0.001	20
72	*Passer rutilans*	Linnaeus, 1758	Russet Sparrow	Jangli chirri	LC	҂	×	×	×	×	҂	×	×	×	҂	21	0.001	20
73	*Pericrocotus cinnamomeus*	Linnaeus, 1758	Small Minivet	Pithpittha	LC	×	×	×	×	×	×	×	×	×	҂	4	0.003	1
74	*Pericrocotus ethologus*	Linnaeus, 1766	Long-tailed Minivet	Raja lal	LC	×	×	×	×	×	×	×	×	×	҂	5	0.003	1
75	*Pericrocotus roseus*	Bangs and Phillips, 1914	Rosy Minivet	Lambi dum, Raja lal	LC	×	×	×	×	×	×	×	×	×	҂	6	0.004	1
76	*Periparus ater*	Temminck, 1836	Coal Tit	Chitti chirri	LC	×	×	×	×	×	×	×	×	×	҂	2	0.001	2
77	*Periparus rubidiventris*	Vieillot, 1818	Rufous-vented Tit	Gulabi Rajalal	LC	×	×	×	×	×	×	×	×	×	҂	3	0.002	2
78	*Periparus rufonuchalis*	Blyth, 1847	Rufous-naped Tit	Pithpittha, pidda	LC	×	×	×	×	×	×	×	×	×	҂	7	0.005	1
79	*Phoenicurus coeruleocephala*	Blyth, 1849	Blue headed or Blue-capped redstart	Pithpittha	LC	×	×	×	×	×	×	×	×	×	҂	2	0.001	1
80	*Phoenicurus ochruros*	Vigors, 1831	Black redstart	Thirtara	LC	×	×	×	×	×	×	×	×	×	҂	2	0.001	1
81	*Phylloscopus collybita*	S.G.Gmelin, 1774	Common Chiffchaff	Thirtara	LC	×	×	×	×	×	×	×	×	×	҂	1	0.001	1
82	*Phylloscopus trochiloides*	Sykes, 1832	Greenish Warbler	Pid-Piddi	LC	҂	×	×	×	×	×	×	×	×	҂	1	0.001	1
83	*Phylloscopus xanthoschistos*	Sundevall, 1837	Grey-hooded Warbler	Heri Piddi	LC	×	×	×	×	×	×	×	×	×	҂	3	0.002	1
84	*Platalea leucorodia*	Gray, 1846	Eurasian Spoonbill	Piddi	LC	×	×	×	×	×	×	×	×	×	҂	2	0.001	1
85	*Prinia gracilis*	Linnaeus, 1758	Graceful Prinia	Dai	LC	×	×	×	×	×	×	×	×	×	҂	5	0.003	1
86	*Prinia hodgsonii*	Lichtenstein, 1823	Grey-breasted Prina	Piddi	LC	×	×	×	×	×	×	×	×	×	҂	2	0.001	1
87	*Prunella fulvescens*	Blyth, 1844	Black-throated Accentor	Piddi	LC	×	×	×	×	×	×	×	×	×	҂	3	0.002	1
88	*Psittacula Himalayana*	Severtsov, 1873	Slaty-headed Parakeet	Kn kali	LC	×	×	×	×	×	×	×	×	×	҂	2	0.001	1
89	*Psittacula krameri*	Desmarest, 1806	Rose-ringed Parakeet	Gani wala tota	LC	×	×	҂	×	҂	×	×	×	×	҂	2	0.001	2
90	*Pteruthius xanthochlorus*	Desmarest, 1806	Green shrike-babbler	Gani wala	LC	×	×	×	×	×	×	×	×	×	҂	4	0.003	1
91	*Ptyonoprogne rupestris*	Gray, 1846	Eurasian Crag Martin	Sehari	LC	×	×	×	×	×	×	×	×	×	҂	3	0.002	1
92	*Pucrasia macrolopha*	Scopoli, 1769	Koklass Pheasant	Mandelli	LC	×	×	×	×	×	҂	×	×	×	҂	60	0.041	3
93	*Pycnonotus leucogenys*	Lesson, 1829	Himalayan Bulbul	Bhaker	LC	×	×	×	×	×	×	×	×	×	҂	35	0.024	1
94	*Pyrrhula aurantiaca*	Gray, 1835	Orange Bullfinch	Finch	LC	×	×	×	×	×	×	×	×	×	҂	5	0.003	1
95	*Rhipidura aureola*	Gould, 1858	Rusty-cheeked Scimitar Babbler	Zangi chrii	LC	×	×	×	×	×	×	×	×	×	҂	2	0.001	1
96	*Saxicola caprata*	Hodgson, 1837	Pied Bush Chat	Piddi	LC	×	×	×	×	×	×	×	×	×	҂	2	0.001	1
97	*Saxicola ferreus*	Linnaeus, 1766	Grey Bush Chat	Salaiti chiri	LC	×	×	×	×	×	×	×	×	×	҂	2	0.001	1
98	*Saxicola torquatus*	Vieillot, 1818	Common Stonechat	Sehari	LC	×	×	×	×	×	×	×	×	×	҂	3	0.002	1
99	*Streptopelia chinensis*	Gray, 1830	Spotted Dove	Kogi	LC	҂	×	×	×	×	҂	×	×	×	҂	45	0.010	9
100	*Streptopelia oriental*	Gray, 1846	Oriental Turtle Dove	Phittli	LC	҂	×	×	×	×	҂	×	×	×	҂	2	0.001	2
101	*Surniculus lugubris*	Latham, 1790	Drongo-cuckoo	Kogi	LC	×	×	×	×	×	҂	×	×	×	҂	6	0.004	1
102	*Tachymarptis melba*	Horsfield, 1821	Alpine Swift	Choti ababil	LC	×	×	×	×	×	×	×	×	×	҂	2	0.001	1
103	*Tarsiger chrysaeus*	Linnaeus, 1758	Golden Bush Robin	Mandli	LC	×	×	×	×	×	×	×	×	×	҂	3	0.002	1
104	*Terpsiphone paradise*	Hodgson, 1845	Asian Paradise Flycatcher	Bori-chiti chirri	LC	×	×	×	×	×	×	×	×	×	҂	55	0.037	1
105	*Trochalopteron variegatum*	Linnaeus, 1758	Variegated Laughingthrush	Taleer	LC	×	×	×	×	×	×	×	×	×	҂	2	0.001	1
106	*Turdoides striata*	Vigors, 1831	Jungle Babbler	Chirii	LC	×	×	×	×	×	҂	×	×	×	҂	7	0.005	1
107	*Turdus atrogularis*	Dumont, 1823	Black-throated Thrush	Jungli chira	LC	×	×	×	×	×	×	×	×	×	҂	31	0.021	1
108	*Turdus boulboul*	Jarocki, 1819	Grey-winged Blackbirds	Chirra	LC	×	×	×	×	×	×	×	×	×	҂	3	0.002	1
109	*Turdus naumanni*	Latham, 1790	Neumann’s or Dusky Thrush	Jangli chira	LC	×	×	×	×	×	×	×	×	×	҂	2	0.001	1
110	*Turdus unicolor*	Temminck, 1820	Tickell’s Thrush	Kholchor	LC	×	×	×	×	×	×	×	×	×	҂	3	0.002	1
111	*Turdus viscivorus*	Tickell, 1833	Mistle Thrush	Kholchora	LC	҂	×	×	×	×	×	×	×	×	҂	2	0.001	2
112	*Upupa epops*	Linnaeus, 1758	Common Hoopoe	Chirii	LC	҂	×	×	×	×	×	×	×	×	҂	11	0.001	2
113	*Urocissa flavirostris*	Linnaeus, 1758	Yellow-billed Blue Magpie	Hud hud,	LC	×	×	×	×	×	×	×	×	×	҂	44	0.030	1
114	*Zoothera dauma*	Latham, 1790	Scaly or White’s or Golden Mountain Thrush	Kholchora	LC	×	×	×	×	×	×	×	×	×	҂	3	0.002	1
115	*Zoothera mollissima*	Latham, 1790	Plain-backed Thrush	Chatri chirri	LC	×	×	×	×	×	×	×	×	×	҂	2	0.001	1
116	*Zosterops palpebrosus*	Blyth, 1820	Oriental White-eye	Chiti akh chiri	LC	×	×	×	×	×	×	×	×	×	҂	35	0.024	1
Mammals																		
117	*Bandicota bengalensis*	Gray, 1835	Indian Mole Rat	Fasli chuha	LC	×	×	×	×	×	×	҂	×	×	×	5	0.021	5
118	*Canis aureus*	Linnaeus, 1758	Asiatic Jackal	Gedar	LC	×	×	×	×	×	×	҂	×	×	×	4	0.017	2
119	*Eoglaucomys fimbriatus*	Gray, 1837	Small Kashmir Flying Squirrel	Chhoti kneez	LC	҂	×	×	×	×	×	҂	×	×	×	8	0.033	5
120	*Funambulus pennantii*	Wroughton, 1905	Northern Palm Squirrel	Gulari	LC	҂	×	×	×	×	×	҂	×	×	×	8	0.033	5
121	*Herpestes auropunctatus*	Hodgson, 1836	Small Indian Mongoose	Satrenga	LC	҂	×	×	×	҂	×	×	×	×	×	5	0.008	5
122	*Herpestes edwardsii*	É.Geoffroy Saint-Hilaire, 1818	Indian Grey Mongoose	Murda tng, Ghorsal	LC	×	×	×	×	҂	×	×	×	×	×	20	0.083	3
123	*Hystrix indica*	Kerr, 1792	Indian Crested Porcupine	Kandy wali segh	LC	҂	×	×	×	×	×	҂	҂	×	×	12	0.050	5
124	*Macaca mulatta*	Zimmermann, 1780	Rhesus Monkey	Buja	LC	×	×	×	×	×	×	҂	×	×	×	5	0.021	2
125	*Manis crassicaudata*	Geoffroy, 1803	Indian Pangolin	Sippa	EN	҂	×	҂	×	×	×	×	҂	҂	×	6	0.025	4
126	*Martes flavigula*	Boddaert, 1785	Yellow-throated Marten	Tobra	LC	×	×	×	×	×	×	×	×	×	×	2	0.008	2
127	*Mus musculus*	Linnaeus, 1758	House Mouse	Ghr ka choha	LC	҂	×	×	×	×	×	҂	×	×	×	7	0.029	6
128	*Ochotona roylei*	Ogilby, 1839	Royle’s pika	Gor ghichoo	LC	×	×	×	×	×	×	҂	×	×	×	40	0.166	4
129	*Paguma larvata*	C.E.H.Smith, 1827	Himalayan Palm Civet	Lak truta	LC	×	×	×	×	×	×	×	×	×	×	44	0.183	2
130	*Panthera pardus*	Kerr, 1792	Common Leopard	Cheeta	VU	×	×	×	×	×	×	҂	×	×	҂	7	0.029	7
131	*Petaurista petaurista*	Pallas, 1766	Giant red Himalayan Flying Squirrel	Bhari kneez	LC	×	×	×	×	×	×	҂	×	×	×	5	0.021	4
132	*Prionailurus bengalensis*	Kerr, 1792	Leopard Cat	Jungli billi	LC	҂	×	×	×	×	×	҂	×	҂	҂	30	0.124	3
133	*Suncus etruscus*	Savi, 1822	Mediterranean Pygmy Shrew	Kera, Chota choha	LC	×	×	×	×	×	×	×	×	×	×	5	0.021	2
134	*Suncus murinus*	Linnaeus, 1766	House Shrew	Anna choha	LC	҂	×	×	×	×	×	҂	×	҂	×	7	0.029	2
135	*Sus scrofa*	Linnaeus, 1758	Indian Wild Boar	Barla	LC	҂	×	×	×	×	×	҂	×	×	×	33	0.012	3
136	*Vulpes vulpes*	Linnaeus, 1758	Red Fox	Rati lumri	LC	҂	҂	×	×	×	×	҂	×	×	×	5	0.021	5

FC (frequency of citation), RFC (relative frequency of citation), CU (cultural uses), MD (medicinal uses), NR (narrative uses), CC (commercial uses), TL (tool uses), ET (entertainment uses), FD (food), HF (harmful), MG (magic), EX (export), OR (ornamental uses), ҂ (yes) and × (no).

**Table 2:** Ethnopharmacological application of avian and mammalian species in ANP, KPK, Pakistan.

Sr. No.	Common Name	Parts Used	Applications	Ailments	Codes	Frequency of Citation	Informants of Major Ailments	Number of Diseases	Fidelity Level	Relative Popularity Level	Rank Order Priority
Birds											
1	Common Myna	M	O	Whooping cough	CM-WC	81	5	2	6.17	1.00	6.17
				Weakness	CM-WN		5		6.17	1.00	6.17
2	Jungle Myna	M	O	Whooping cough	JM-WC	45	7	2	15.56	0.89	13.89
				Weakness	JM-WN		5		11.11	0.89	9.92
3	Spotted Dove	M	O	Maturity in girls	SP-MG	45	9	1	20.00	0.89	17.86
4	House Crow	B	T	Ear infection	HC-EI	18	8	1	44.44	0.36	15.87
5	Large Cuckooshrike	WB	O	Malaria	LCS-MR	7	2	1	28.57	0.14	3.97
6	Mistle Thrush	M, FE	O	Skin diseases	MT-SD	2	2	1	100	0.04	3.97
7	Hill Pigeon	M, E	O	Menstrual bleeding	HP-MR	30	12	8	40.00	0.60	23.81
				Respiratory problems	HP-BC		14		46.67	0.60	27.78
				Girls’ puberty	HP-PG		5		16.67	0.60	9.92
				Joint pain	HP-PL		3		10.00	0.60	5.95
				Seizure disorder	HP-EL		2		6.67	0.60	3.97
				Low hemoglobin	HP-AM		5		16.67	0.60	9.92
				Impaired fecundity	HP-IF		3		10.00	0.60	5.95
				Low blood pressure	HP-BP		5		16.67	0.60	9.92
8	Common Pigeon	M, E	O	Menstrual bleeding	CM-MR	55	12	8	21.82	1.00	21.82
				Respiratory problems	CM-BC		14		25.45	1.00	25.45
				Girls’ puberty	CM-PG		5		9.09	1.00	9.09
				Joint pain	CM-PL		3		5.45	1.00	5.45
				Seizure disorder	CM-EL		2		3.64	1.00	3.64
				Low hemoglobin	CM-AM		5		9.09	1.00	9.09
				Impaired fecundity	CM-IF		3		5.45	1.00	5.45
				Low blood pressure	CM-BP		5		9.09	1.00	9.09
9	Kalij Pheasant	M	O	Weakness	KP-WN	60	3	2	5.00	1.00	5.00
				Paralysis	KP-PL		3		5.00	1.00	5.00
10	House Sparrow	M, E	O	Weakness	HS-WN	62	20	3	32.26	1.00	32.26
				Fever	HS-FV		12		19.35	1.00	19.35
				Low blood pressure	HS-BP		11		17.74	1.00	17.74
11	Russet Sparrow	M	O	Weakness	RS-WN	21	20	2	95.24	0.42	39.68
				Fever	RS-FV		11		52.38	0.42	21.83
12	Greenish Warbler	M	O	Fever	GW-FV	1	1	3	100	0.02	1.98
				Paralysis	GW-PL		1		100	0.02	1.98
				Infertility	GW-IF		1		100	0.02	1.98
13	Grey-hooded Warbler	M	O	Fever	GHW-FV	3	1	3	33.33	0.06	1.98
				Paralysis	GHW-PL		1		33.33	0.06	1.98
				Infertility	GHW-IF		1		33.33	0.06	1.98
14	Green shrike-babbler	M	O	Fever	GSB-FV	4	1	3	25.00	0.08	1.98
				Paralysis	GSB-PL		1		25.00	0.08	1.98
				Infertility	GSB-IF		1		25.00	0.08	1.98
15	Koklass Pheasant	M	O	Weakness	GSB-WN	60	3	2	5.00	1.00	5.00
				Paralysis	KP-PL		2		3.33	1.00	3.33
16	Oriental Turtle Dove	M	O	Maturity in girls	KP-MG	2	2	1	100	0.04	3.97
17	Jungle Babbler	M	O	Fever	JB-FV	7	1	3	14.29	0.14	1.98
				Paralysis	JB-PL		1		14.29	0.14	1.98
				Infertility	JB-IF		1		14.29	0.14	1.98
18	Common Hoopoe	M	O	Kidney problems	CH-KP	11	2	1	18.18	0.22	3.97
**Mammals**											
19	Leopard Cat	F	T	Joint pain	LC-JP	30	3	1	10.00	0.60	5.95
20	House Shrew	F	T	Scrotal swelling	HS-SS	7	4	3	57.14	0.14	7.94
				Snake bite	HS-SB		3		42.86	0.14	5.95
				backbone pain	HS-BB		3		42.86	0.14	5.95
21	Northern Palm Squirrel	M	T	Epilepsy	NPS-EL	8	5	1	62.50	0.16	9.92
22	Indian Crested Porcupine	F	T	Skin infection	ICP-SI	12	5	2	41.67	0.24	9.92
				Rheumatic pain	ICP-RP		5		41.67	0.24	9.92
23	Indian Pangolin	S, M	T	Feet swelling	IP-FS	6	7	2	116.7	0.12	13.89
				Sexual power	IP-SP		4		66.67	0.12	7.94
24	House Mouse	F	T	Arthritis	HM-AR	7	4	2	57.14	0.14	7.94
				Analgesic	HM-AL		3		42.86	0.14	5.95
25	Small Indian Mongoose	F	T	Herpes	SIM-HP	5	4	3	80.00	0.10	7.94
				Joint pain	SIM-JP		3		60.00	0.10	5.95
				Backbone pain	SIM-BB		3		60.00	0.10	5.95
26	Red Fox	F, M	T	Ear pain	RF-EP	5	3	3	60.00	0.10	5.95
				Joint pain	RF-JP		3		60.00	0.10	5.95
				Epilepsy	RF-EL		3		60.0	0.10	5.95
27	Indian Wild Boar	F	T	Paralysis	IWB-PL	33	3	2	9.09	0.65	5.95
				Burn	IWB-BR		3		9.09	0.65	5.95
28	Small Kashmir Flying Squirrel	F	T	Joint pain	SKFS-JP	8	2	3	25.00	0.16	3.97
				Backbone pain	SKFS-BB		2		25.00	0.16	3.97
				Sexual power	SKFS-SP		1		12.50	0.16	1.98
29	Giant Red Himalayan Flying Squirrel	F	T	Sexual power	GFKS-SP	5	3	1	60.00	0.10	5.95
30	Indian Grey Mongoose	F	T	Herpes	IGM-HS	20	3	3	15.00	0.40	5.95
				Joint pain	IGM-JP		3		15.00	0.40	5.95
				Backbone pain	IGM-BB		3		15.00	0.40	5.95
31	Himalayan Palm Civet	F	T	Joint pain	HPC-JP	44	1	3	2.27	0.87	1.98
				Backbone pain	HPC-BB		1		2.27	0.87	1.98
				Sexual power	HPC-SP		2		4.55	0.87	3.97
32	Common Leopard	F	T	Sexual power	CL-SP	7	5	1	71.43	0.14	9.92

Parts used: F (at), S (scale), M (meat), E (egg), FE (feather), B (bone), WB (whole body), O (oral), and T (topical).

## Data Availability

The data are available on request to the first author.
